# ASO Author Reflections: Palliative Therapy Might be an Alternative When the Risks of Surgery for Perihilar Cholangiocarcinoma are High

**DOI:** 10.1245/s10434-024-15830-8

**Published:** 2024-07-16

**Authors:** Pim B. Olthof, Anne-Marleen van Keulen, Stefan Buettner, Bas Groot Koerkamp

**Affiliations:** 1 Department of Surgery, Erasmus MC Cancer Institute, Rotterdam, The Netherlands; 2 Department of Surgery, Cancer Center Amsterdam, Amsterdam UMC, University of Amsterdam, Amsterdam, The Netherlands; 3 Department of Surgery, University Medical Center Groningen, University of Groningen, Groningen, The Netherlands

## Past

Surgical resection can offer long-term survival to patients with perihilar cholangiocarcinoma, with a median overall survival rate of 30 months.^1^ Several negative prognostic factors can influence survival. Positive resection margins are associated with median survival of 21 months, and when lymph nodes are also present, median survival is 12 months.^2^ In patients who underwent palliative systemic therapy, median overall survival was also 12 months.^1^ While surgical resection is the goal for most patients with perihilar cholangiocarcinoma, the comparable survival associated with palliative systemic therapy and surgery in high-risk patients questions the benefit of surgery for some patients.

## Present

Using data from the European Network for the Study of Cholangiocarcinoma (ENS-CCA) registry, 92 patients who underwent palliative systemic therapy were compared with 146 patients who underwent resection and had postoperative positive resection margins. Median overall survival was comparable between both groups (17.1 vs. 16 months; *p* = 0.06), while 5-year overall survival was 20.0% with R1 resection and 2.2% with chemotherapy. Negative prognostic factors were more frequent in the chemotherapy group and type of treatment (i.e., R1 resection or palliative chemotherapy) was not an independent predictor of overall survival in multivariable analysis.^3^

## Future

A randomized trial comparing palliative treatment with resection in patients with high-risk perihilar cholangiocarcinoma has not been performed and would be challenging. The present study shows that the benefit of surgery should be carefully weighted against the substantial risks of surgery in these patients. Major liver resection is often required and survival can be limited and even similar to palliative treatment without surgery. Surgery however can still offer long-term survival in a small proportion of patients. Surgery remains the standard for resectable perihilar cholangiocarcinoma. When the anticipated risk of positive margins is high and the risk of postoperative morbidity and mortality is considerable, palliative systemic therapy should be considered.^4^
